# Brain sites involved in endogenous analgesia

**DOI:** 10.1097/j.pain.0000000000004101

**Published:** 2026-11-02

**Authors:** Luke A. Henderson, Lewis S. Crawford, James W.M. Kang, Kevin A. Keay

**Affiliations:** a School of Medical Sciences (Neuroscience), University of Sydney, Australia; b Brain and Mind Centre, University of Sydney, Australia

## Abstract

Pain modulation is a fundamental survival mechanism that prioritises, shapes, and suppresses nociceptive inputs to optimise behaviour. Although acute pain signals potential or actual tissue damage, sustained pain can impair essential behaviours, necessitating modulatory circuits that dynamically regulate nociception according to context and motivational state. These systems are deeply conserved, embedded within primitive spinal and brainstem structures, and capable of powerful modulation independent of cortical input, although higher regions such as the prefrontal cortex can influence their function. Decades of work have sought to identify these core modulatory circuits to advance basic understanding and develop strategies for clinically meaningful hypoalgesia. More recently, dysfunction within these circuits has been implicated in chronic pain, driving efforts to characterise how their organisation and function change during persistent pain states. Although most mechanistic insights come from animal studies, human neuroimaging, particularly since the advent of functional magnetic resonance imaging, has begun to map their location and engagement during pain modulation. Early work focused on forebrain regions due to the limited spatial resolution of standard MRI, but ultra-high-field functional magnetic resonance imaging now enables mm^3^-scale investigation of brainstem nuclei. This review synthesises evidence from animal and human studies to outline the best-characterised modulatory pathway, consisting of projections from the midbrain periaqueductal gray to the rostral ventromedial medulla and onward to the dorsal horn/spinal trigeminal nucleus, and highlights recent work detailing its organization.

## 1. Introduction

Pain modulation is a key survival mechanism that prioritises, shapes, or suppresses nociceptive input to optimise behaviour. Although acute pain signals potential tissue damage, excessive or inflexible responses can hinder defensive behaviours, feeding, and reproduction. To address this, evolution has favoured modulatory circuits that dynamically regulate nociception based on context, motivation, and behavioural demands. Although higher-order forebrain regions (eg, prefrontal, cingulate, and motor cortices, amygdala, and nucleus accumbens) are known to influence pain, it is their projections—direct or indirect—to brainstem regions, which in turn project to the spinal cord and brainstem, that ultimately control incoming noxious information.^35,37^

Preclinical studies show that pain modulation is mediated by a distributed brainstem network, including the midbrain periaqueductal gray (PAG), nucleus cuneiformis, locus coeruleus, A5 cell group, rostral ventromedial medulla (RVM), and subnucleus reticularis dorsalis, which influence incoming nociceptive transmission.^13,16,21,23,53,55,71,93,111,132^ Although regulated by higher centres, these regions can independently modulate nociceptive signalling, underscoring their role as core components of an evolutionarily conserved system. Their localisation within phylogenetically ancient structures, the spinal cord and brainstem, highlights pain regulation as a fundamental survival function. Accordingly, extensive research has characterised their organisation and mechanisms, both to understand basic biology and to harness them for analgesia. More recently, dysfunction in these systems has been linked to chronic pain, driving efforts to restore normal circuit function.

Although much of our mechanistic understanding derives from experimental animal studies, advances in human neuroimaging have enabled in vivo investigation of these circuits. Since the introduction of functional magnetic resonance imaging (fMRI) in the early 1990s,^120^ numerous studies have identified activity changes in brain regions associated with pain modulation across paradigms such as placebo analgesia, conditioned pain modulation, and offset analgesia.^5,18,21,33,34,153,166,168,169^ The vast majority of these studies focused on forebrain changes, as the brainstem is small and its individual nuclei even smaller, making it difficult to accurately explore brainstem sites with the spatial resolution afforded by fMRI at standard MRI field strengths (eg, 1.5 and 3 T). However, more recent ultra-high-field fMRI studies (7 T) are now exploring brainstem circuits with mm^3^ specificity and are beginning to detail the function of various brainstem nuclei during painful stimuli and pain-modulatory tasks.^134^

Although pain modulation arises from a distributed network of brainstem circuits, in this review, we focus on the currently best-characterised pain-modulatory pathway, the PAG-RVM-dorsal horn (DH)/spinal trigeminal (SpV) circuit, drawing on evidence from both experimental animal and recent ultra-high-field human fMRI studies. A clear mechanistic map of this pain-modulatory pathway is essential for understanding the pathophysiology of chronic pain, enabling investigators to identify which components of the system become dysregulated and which circuit elements may represent targets for restoring normal pain-modulatory control.

## 2. Periaqueductal gray and defensive behaviours

A fundamental requirement for all living organisms is the ability to keep their physical structure intact and functioning. When an organism experiences pain, signalling risk to body integrity, the course of action selected reflects a combination of reflexive complex behavioural reactions shaped by rapid subconscious evaluation of the context. Among the most frequent and well-documented behavioural reactions to pain is the classic “fight or flight” defence response, in which an organism either confronts a source of stress, threat, or pain or attempts to escape it. The deeply rooted evolutionary priority of survival requires the effective execution of these behaviours, and therefore it is not surprising that the circuitry critical for the effective expression of these responses lies within the brainstem^14,99^ and that these integrated behaviours can be expressed without the need for inputs originating above the brainstem level.^31,128^

Experimental animal studies have identified core circuits for defensive behaviour. Early work in decorticate cats showed the hypothalamus is necessary for “rage-like” defensive behaviours^11,12,73^ leading to a “hypothalamic defence area” in dorsomedial, ventromedial, and perifornical regions.^59,158^ This was later expanded to include the amygdala, bed nucleus of the stria terminalis, and PAG.^51,78^ Evidence indicates a hierarchical pathway in which the PAG is central: Its lesions abolish effects of upstream stimulation, while its activation still evokes defence after upstream lesions, and it is critical for natural defensive behaviours.^51,78^ Importantly, hypothalamic lesions lower defensive thresholds,^45,47^ whereas PAG activation produces coordinated autonomic, vocal, and motor responses.^30^ hypothalamic lesions do not impair spontaneous defensive behaviours,^45,47^ whereas PAG activation produces coordinated autonomic, vocal, and motor responses.^30^ Together, these findings identify the PAG as the core defensive circuit, modulated by higher regions.

Active, fight-flight defensive behaviours are most often elicited by activation of neurons in the region immediately lateral to the cerebral aqueduct. More specifically, microinjections of excitatory amino acids in freely moving rats revealed that neurons of the rostral lateral PAG (lPAG) integrate confrontational, “fight” defensive behaviours characterized by facing towards and backing away from a threatening stimulus, whereas neurons of the caudal lPAG integrate “flight” defensive behaviours characterized by escape and avoidance of the stimulus.^41^ These behavioural responses are coupled to cardiovascular changes supporting their execution, with rostral lPAG stimulation increasing blood flow to the head and face and caudal lPAG stimulation increasing blood flow directed to the hindlimbs.^29^

A substantial body of work has further refined the functional organisation of lPAG circuits, particularly in relation to their distinct forebrain inputs and roles in defensive control. Converging anatomical and optogenetic studies demonstrate that both prefrontal and cingulate regions have distinct projections into the lPAG, selectively regulating the expression of active defensive/coping behaviours. There is compelling evidence that indicates that prefrontal-PAG pathways bidirectionally regulate defensive coping styles, with increased prefrontal drive promoting active defensive behaviours and suppressing vlPAG-mediated functions, highlighting the principles of top-down control in the modulation of defensive states.^84,94,126,130^ These preclinical animal studies dissecting circuit-level organisation align with human neuroimaging findings demonstrating a shift from prefrontal to PAG dominance as threat imminence increases, consistent with a transition from deliberative to reflexive control.^112,140^ In this context, emerging work emphasizes that distinct PAG circuits differentially support reflexive “panic,” vs planned escape, reinforcing the view that the PAG operates within a broad, hierarchically organized network integrating cognitive appraisal with brainstem defensive outputs.

In addition to the rostrocaudal functional differentiation in the lPAG, excitation of neurons in the PAG ventrolateral to the aqueduct (vlPAG) evoked a reaction characterized by decreased spontaneous activity and decreased reactivity to surrounding stimuli, this response was coupled with decreases in blood pressure and heart rate.^28,40,170^ The vlPAG is a core neural substrate for passive emotional coping responses, mediating behavioral immobility and quiescence when threats are inescapable rather than reflecting behavioural “failure.”^86,87^ In preclinical models, c-Fos mapping and pharmacological inactivation studies have demonstrated that the vlPAG is necessary for both conditioned freezing and the suppression of active defense. The vlPAG is also critical for postencounter freezing and postthreat recovery, whereby quiescence is initiated after significant threat or danger has passed.^154^ These passive defensive states are driven by both cholinergic and opioidergic signalling.^113–115^ These postthreat behaviours have also been observed in people and attributed to vlPAG function.^150^

## 3. Periaqueductal gray and deep/inescapable vs superficial/escapable pain

In a series of parallel studies, using the expression of the immediate early gene *c-fos* as an indicator of neural activity, it was revealed that painful radiant heat applied to the skin preferentially activated contralateral lPAG neurons, whereas muscle pain induced by carrageenan or formalin injections, joint pain induced by kaolin and carrageenan, and visceral/cardiac pain elicited by intraperitoneal acetic acid or intravenous serotonin preferentially activated neurons in the contralateral vlPAG region.^88^ In addition, anatomical tract tracing studies showed that the neurons in the SpV, which receive noxious information from the head, project primarily to the contralateral rostral lPAG, whereas neurons in the superficial laminae of the DH, which receive noxious inputs from the body, project to the contralateral caudal lPAG. In addition, neurons in the deep and superficial DH and the nucleus of the solitary tract, which receive primarily noxious information from muscle and viscera, project primarily to the contralateral vlPAG without any obvious somatotopic organization.^9,89,159^ Data from both the c-Fos and tract tracing studies are consistent with the idea that cutaneous pain activates the lPAG, which, when stimulated, evokes active defensive behaviours, ie, flight and fight, supported by increased heart rate and blood pressure; whereas, pain originating in deeper tissues such as muscle and viscera activates the vlPAG, which, when stimulated, evokes more passive defensive behaviours characterised by quiescence and hyporeactivity, accompanied by decreased blood pressure and heart rate.^8,41^

These experimental findings are consistent with clinical observations made over 80 years ago by Sir Thomas Lewis, who noted that cutaneous pain triggered quick-protective reflexes, tachycardia and invigorating sensations, whereas pain derived from muscle or deeper structures elicited quiescence and social disengagement coupled with bradycardia and hypotension.^68^ These studies led to the concept that the PAG contains multiple longitudinally oriented columns, with the lPAG column critical for active defensive behaviours and the vlPAG column critical for passive defensive behaviours (Fig. 1). More recent human studies suggest that the relationship between the tissue location of a noxious stimulus and autonomic changes may not be straightforward. For example, both subcutaneous hypertonic saline injections (cutaneous pain) and intramuscular hypertonic saline injections (muscle pain) have been reported to evoke similar increases in muscle sympathetic nerve activity (MSNA), blood pressure, and heart rate, with no clear differential effect between skin and muscle pain.^25^ In contrast, tonic (45-minute) intramuscular hypertonic saline produces 2 stable response patterns across individuals, either sustained increases or sustained decreases in MSNA, blood pressure, and heart rate.^50,91^ Direct electrical stimulation of the PAG in humans appears more consistent with preclinical findings: A seminal study demonstrated that ventrolateral PAG (vlPAG) stimulation decreases sympathetic burst frequency and blood pressure, whereas more dorsal, likely dorsolateral PAG (dlPAG), stimulation shifts burst amplitudes toward larger bursts without altering blood pressure.^142^ Similarly, an earlier study reported that PAG stimulation can evoke either increases or decreases in blood pressure and heart rate depending on the site of stimulation.^64^

**Figure 1.**
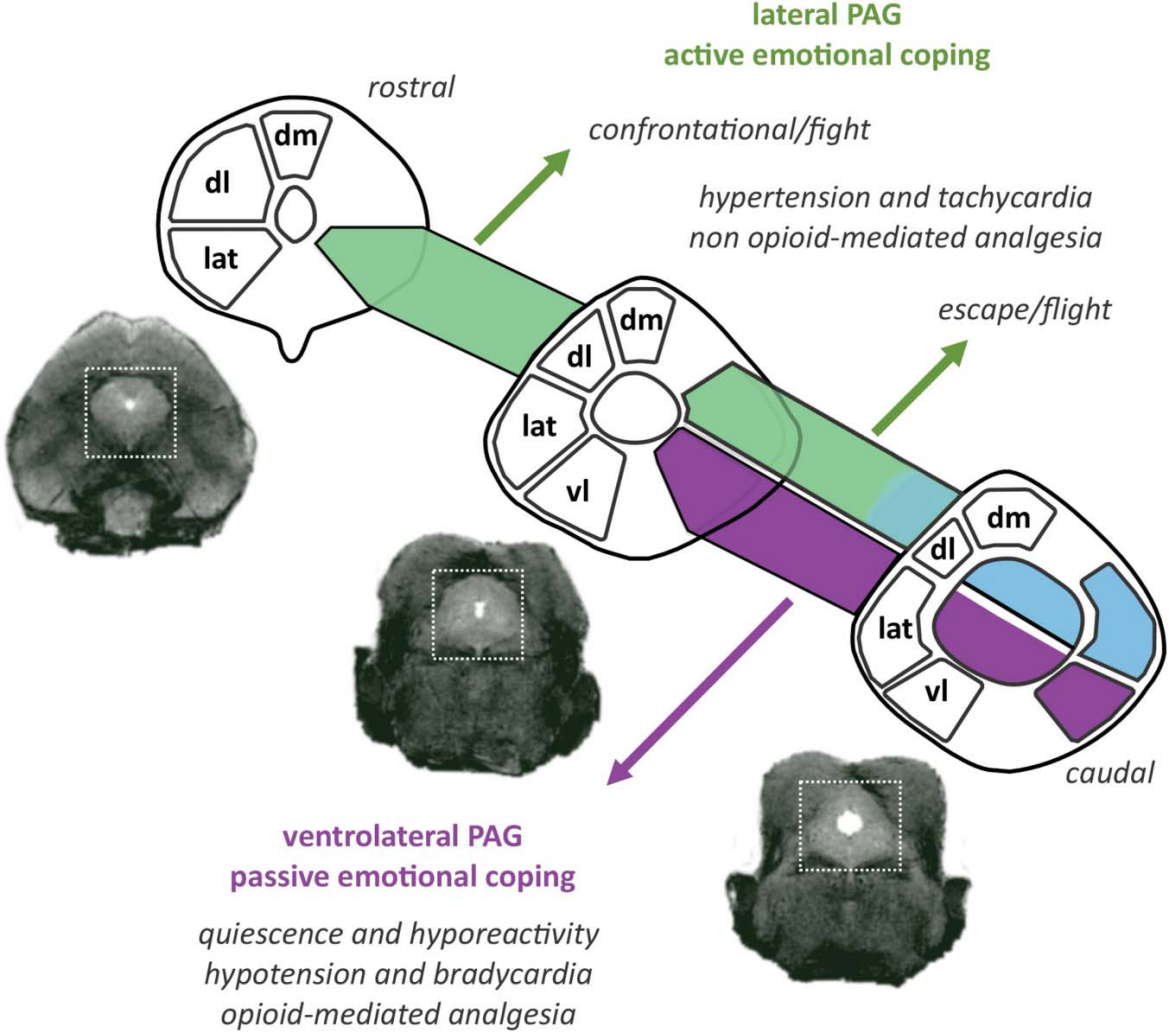
Illustration of the dorsomedial (dm), dorsolateral (dl), lateral (lat), and ventrolateral (vl) columns of the PAG. The location of the PAG is indicated on a high-resolution T2-weighted anatomical magnetic resonance imaging scan of a postmortem rat. Stimulation of the lPAG evokes active emotional coping strategies including defensive reactions (fight rostrally, flight caudally), hypertension, tachycardia, and a nonopioid-mediated analgesia. By contrast, stimulation of the vlPAG evokes passive emotional coping strategies characterised by quiescence, hyporeactivity, hypotension, bradycardia, and an opioid-mediated analgesia. lPAG, lateral periaqueductal gray; PAG, periaqueductal gray; vlPAG, ventrolateral periaqueductal gray.

Shortly after the initial delineation of the columnar organization of the PAG, it was observed that while brief and intermittent noxious cutaneous stimuli activated the lPAG, more prolonged noxious cutaneous stimuli, along with muscle pain, preferentially activated the vlPAG^87^ (Fig. 2A). This led to the more nuanced and current view that the representation of pain in the PAG reflects behavioural significance rather than tissue origin, ie, the lPAG mediates behavioural responses to escapable noxious stimuli, whereas the vlPAG mediates behavioural responses to inescapable noxious events.

**Figure 2.**
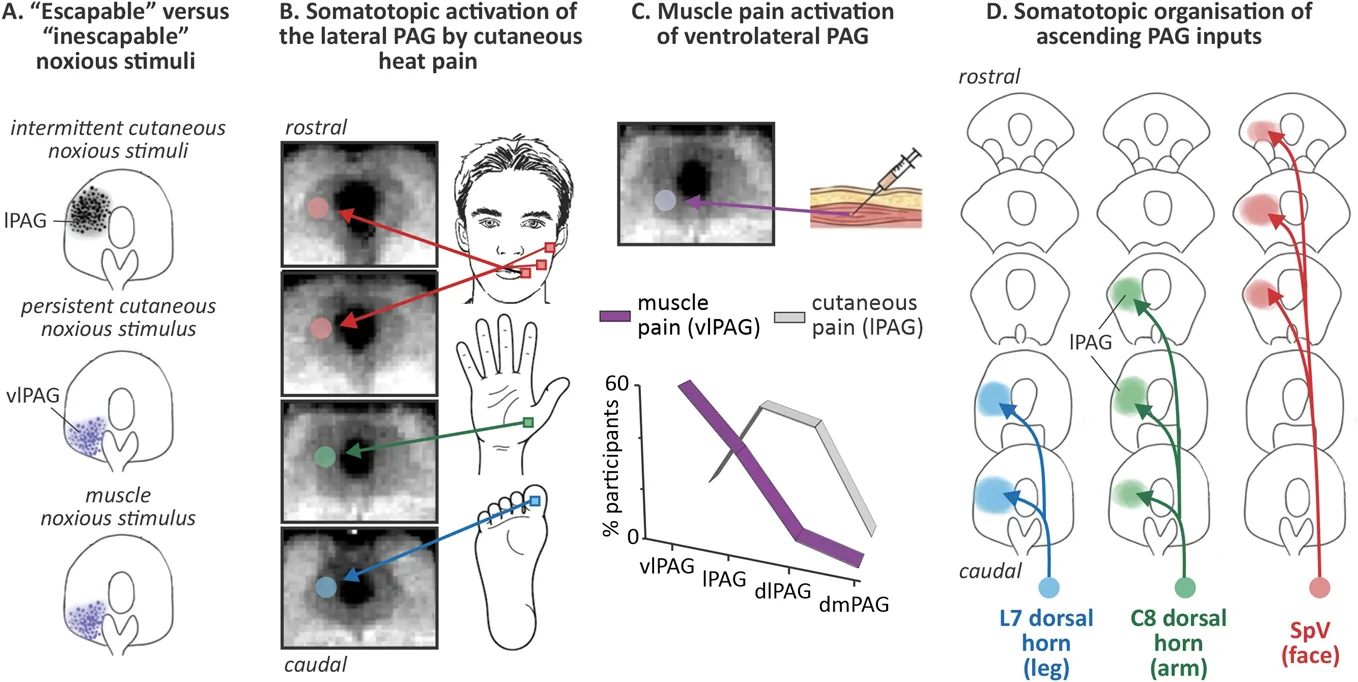
(A) Schematic representative coronal section through the midbrain periaqueductal gray matter (PAG) showing the locations of Fos-like immunoreactive neurones in the lateral PAG (lPAG) and ventrolateral PAG (vlPAG) during intermittent noxious cutaneous stimuli, persistent noxious cutaneous stimuli and muscle noxious stimuli; adapted from 87. (B) Somatotopic organization of functional magnetic resonance imaging (fMRI) signal intensity changes in the lPAG during noxious cutaneous heat stimuli applied to 3 locations on the face, the hand and the foot; adapted from 146. (C) Location of fMRI signal intensity changes during forearm muscle pain. The plot below shows the activation patterns of each PAG column during and muscle pain stimuli. dlPAG, dorsolateral PAG; dmPAG, dorsomedial PAG, adapted from 146. (D) Schematic representative coronal sections through the rostrocaudal extent of the PAG showing a somatotopic organization of ascending afferent inputs from the lumbar 7 (L7) dorsal horn, cervical 8 (C8) dorsal horn and spinal trigeminal nucleus (SpV) to the contralateral lPAG; adapted from 159.

Recently, we have used ultra-high-field fMRI to determine whether this columnar PAG organization is conserved in humans. Using intermittent noxious thermal stimulation of the foot, hand, and 3 locations on the face to elicit brief cutaneous pain, as well as injections of hypertonic saline into the flexor carpi radialis muscle in the forearm to elicit prolonged muscle pain, we mapped fMRI signal intensity changes in the PAG in 25 pain-free control participants.^146^ Consistent with the PAG columnar organisation described in experimental animal studies, we found that brief cutaneous pain preferentially activated the lPAG contralateral to stimulation, whereas prolonged muscle pain preferentially activated the contralateral vlPAG (Figs. 2B and C). Furthermore, we found that cutaneous painful stimuli applied to the arm and leg activated the caudal lPAG, whereas pain applied to the face activated the rostral lPAG (Fig. 2B). Although a subsequent study reported a somatotopic organization for noxious facial stimuli,^107^ we found that the lPAG did not have a fine-grained somatotopic organisation; rather, noxious stimuli of each of the 3 different regions of the face activated the same rostral lPAG region, and similarly, noxious stimulation of the arm and leg activated the same caudal lPAG region. Although this relatively coarse somatotopy within the lPAG could reflect residual limitations of fMRI in resolving finer-grained organisation, it may also indicate convergence of inputs from multiple body regions onto a common region within the lPAG, but whether this involves convergence onto similar populations of neurons remains unknown. Indeed, consistent with a crude lPAG somatotopic organization, anatomical studies in rats, cats, and primates have revealed that neurons in the SpV project to the rostral lPAG and those in the DH of the spinal cord project to the intermediate and caudal lPAG^89,159^ (Fig. 2D). Such a coarse somatotopic organisation is consistent with the broader functional logic of the lPAG, ie, that it does not require high-precision localisation of noxious stimuli, only a general body vs face discrimination, to rapidly initiate appropriate fight-or-flight responses.

## 4. Periaqueductal gray and hypoalgesia

Although the activation of pathways carrying noxious inputs to the PAG is critical for initiating active defensive behavioural reactions, an accompanying hypoalgesia permits these defensive responses to proceed without interruption by other less “urgent” noxious or nonnoxious somatosensory inputs. Similarly, a hypoalgesic state provides a key advantage for the expression of passive defensive behaviours, allowing an organism to remain quiescent and withdrawn from its environment without interruption by either ongoing noxious inputs or new and less significant noxious or nonnoxious somatosensory inputs. Much of the research focussed on the critical role of the PAG in integrating defensive behaviours studies was being conducted contemporaneously with studies exploring the role of the PAG in modulating pain intensity. In the late 1960s it was reported that electrical stimulation of the midbrain adjacent to the aqueduct produced a profound hypoalgesia.^128^ In the years that followed, numerous studies confirmed that stimulation of the PAG in the rat, cat and monkey each produces a profound hypoalgesia.^13,14,105,106,121,138^ These studies indicated that analgesic effects could be elicited from large areas throughout the PAG, although it was noted that measuring analgesic effects could be compromised by strong changes in behaviour at many stimulation sites.^19^

In the early 1980s, systematic mapping of the PAG in freely moving rats revealed that the ventral PAG, particularly the vlPAG, contains sites capable of evoking “pure analgesia,” whereby stimulation increases nociceptive thresholds without producing strong aversive behaviours.^48,49^ In the early 1990s, more specific neural excitation techniques, such as microinjection of excitatory amino acids, were developed which allowed researchers to map more precisely the brainstem sites from which hypoalgesia could be produced. In 1 study, the excitatory amino acid L-glutamate was microinjected at 331 brain sites from the lower medulla to the diencephalon, and tail-flick and hot-plate latencies were measured as indicators of analgesic responses.^83^ It was found that injection sites within the lPAG and vlPAG regions were particularly effective at evoking analgesic responses, many also evoking behavioural agitation that resembled spontaneous pain, characterised by “excitation with motivated running and vocalisation.” In addition, stimulation of the area encompassing the RVM evoked antinociception, however, without the agitation behaviours that occurred during PAG stimulation. Many previous studies had also shown that this RVM region is a potent site for glutamate-evoked hypoalgesia. In several studies spanning the 1970s and 1980s, it was shown that hypoalgesia evoked from both lPAG and vlPAG were mediated by a critical synapse in this RVM region.^2,16,52^

In addition to microinjections of excitatory amino acids, multiple experimental animal studies have reported that injections of opioid agonists into the PAG, specifically into the ventrolateral region, can also evoke a strong hypoalgesia.^79,81,82^ Importantly, opioid injections made into the lPAG do not evoke these analgesic responses. Further highlighting this distinct regional opioid sensitivity in the PAG, studies exploring the effects of opioid antagonists on PAG-evoked hypoalgesia found that ventral PAG-evoked hypoalgesia was significantly attenuated by opioid antagonist treatment, whereas dorsal PAG-evoked hypoalgesia was unaltered.^26,144^ Consistent with the striking anatomical differences in opioid-dependent PAG analgesic effects in experimental animals, several studies reported that stimulation of the PAG in humans evokes a hypoalgesia that can be blocked by opioid antagonist administration, whereas other studies reported that PAG hypoalgesia could not be fully blocked with naloxone, strongly suggesting that the human PAG can also produce both an opioid and nonopioid-mediated hypoalgesia.^77,167^

It is now very well-established that stimulation of the vlPAG evokes an opioid-mediated hypoalgesia and that lPAG evokes a nonopioid hypoalgesia.^9^ The nonopioid hypoalgesia evoked from the lateral PAG has been shown to be mediated by an endocannabinoid-dependent mechanism in which glutamate acting at mGlu5 receptors triggers diacylglycerol lipase-alpha-mediated release of 2-arachidonoylglycerol (2-AG) that acts on presynaptic cannabinoid type 1 (CB1) receptors to reduce inhibition. This produces analgesia primarily through a process of GABA-ergic disinhibition, which suppresses tonic GABAergic inputs and activates PAG output neurons projecting into descending pain-modulatory circuits.^57,65^ This hypoalgesia can also be activated by substance P,^42^ neurotensin,^17^ serotonin,^147^ and dopamine.^143^ The nonopioid lPAG analgesia is driven by multiple neurochemical systems interacting with endocannabinoid signalling and GABA disinhibition.

The lateral and ventrolateral PAG each mediate these analgesic effects through direct projections to the RVM, which in turn modulates incoming noxious information in the DH or SpV.^1,32,53,55,56,69,76,104,127^ Electrophysiological studies have shown that the RVM contains neurons that can inhibit or facilitate noxious inputs^54,69,90^ and that some analgesic responses, for example, those which result from morphine administration, involve activation of inhibitory and inhibition of facilitatory RVM cells, which ultimately reduces nociceptive transmission in the DH or SpV.^70^ Indeed, although it was originally thought that the PAG-RVM-DH/SpV system was exclusively inhibitory,^127,135,136,157^ it is now recognised that this circuit can also act to enhance nociceptive transmission and pain intensity.^66,141,171^ It should be noted that findings derived from exogenous opioid administration may not fully generalize to endogenous opioid signalling, which is likely to be more spatially and temporally restricted. Nevertheless, converging evidence indicates that endogenous opioid peptides within the PAG and RVM contribute to descending modulation of nociception.^7^

## 5. Periaqueductal gray and somatotopic organization for hypoalgesia

The differential behavioural effects of rostral vs caudal lPAG stimulation, coupled with the crude somatotopic organization of ascending afferent inputs and pain activation patterns described above, led us to ask the question whether there is a lPAG somatotopic organization of analgesic effects? As the rostral lPAG is driven by primarily orofacial noxious inputs and mediates confrontation and fight behaviours, expressed primarily through head and neck-related motor outputs, we hypothesised that this lPAG region would mediate a hypoalgesia restricted to the face. In contrast, since the caudal lPAG is activated by noxious inputs arising from the body and mediates escape and flight behaviours, dominated by body-related motor outputs, we hypothesised that this lPAG region would mediate a hypoalgesia restricted to the body. These spatially restricted patterns of hypoalgesia would confer a significant behavioural advantage, since during confrontation and fight behaviours, the lPAG could preferentially inhibit noxious inputs from the face, whereas during escape and flight behaviours it could inhibit noxious inputs preferentially from the body. That is, the lPAG could produce a hypoalgesia in the region most vulnerable during each behaviour. This restricted analgesic control would prevent pain from interrupting the highly integrated and spatially specific defensive behaviours while preserving sensory acuity required for effective threat assessment.

Over recent decades, brain imaging studies have examined the brainstem, particularly the PAG, in human analgesia. Pharmacological fMRI shows that placebo analgesia can depend on an opioid-sensitive descending circuit, with naloxone reducing behavioural and neural responses and disrupting anterior cingulate-PAG coupling.^46^ Behavioural work similarly indicates that expectation-driven placebo effects rely on endogenous opioids, while conditioning can recruit additional nonopioid mechanisms, producing responses only partially or not reversed by naloxone.^3^ Positron emission tomography studies further demonstrate placebo-induced μ-opioid release in the PAG and adjacent midbrain, correlating with pain relief and increased PAG–anterior cingulate connectivity.^152^ Meta-analyses confirm robust PAG involvement during expectation and nociceptive processing,^4^ and recent findings suggest PAG activity tracks the precision of prior expectations, consistent with Bayesian models of pain modulation.^63^ Despite this, technical limitations have prevented resolution of the PAG's columnar or somatotopic organization.

To determine whether the lPAG is somatotopically organized with respect to hypoalgesia, we recently evoked hypoalgesic responses in different body regions using a spatially restricted, topical cream-based placebo paradigm and measured brainstem activity using ultra-high-field fMRI.^36^ In our recent study, we deceptively applied different intensity thermal stimuli onto sites on the face (n = 45), arm (n = 46) and/or leg (n = 41) to condition pain-free participants to believe that application of a placebo cream (sham “lidocaine”) was acting to reduce their pain relative to the adjacent application of control cream (“vaseline”). In a subsequent session, while collecting ultra-high-field (7-T) fMRI, we applied identical intensity stimuli to both cream sites (“vaseline”/control; “lidocaine”/placebo) and recorded perceived pain intensity. We classified participants as either responders if they showed a significant reduction in pain intensity reporting or nonresponders for each stimulus site and determined the changes in signal intensity associated with evoking analgesic responses (Fig. 3A).

**Figure 3.**
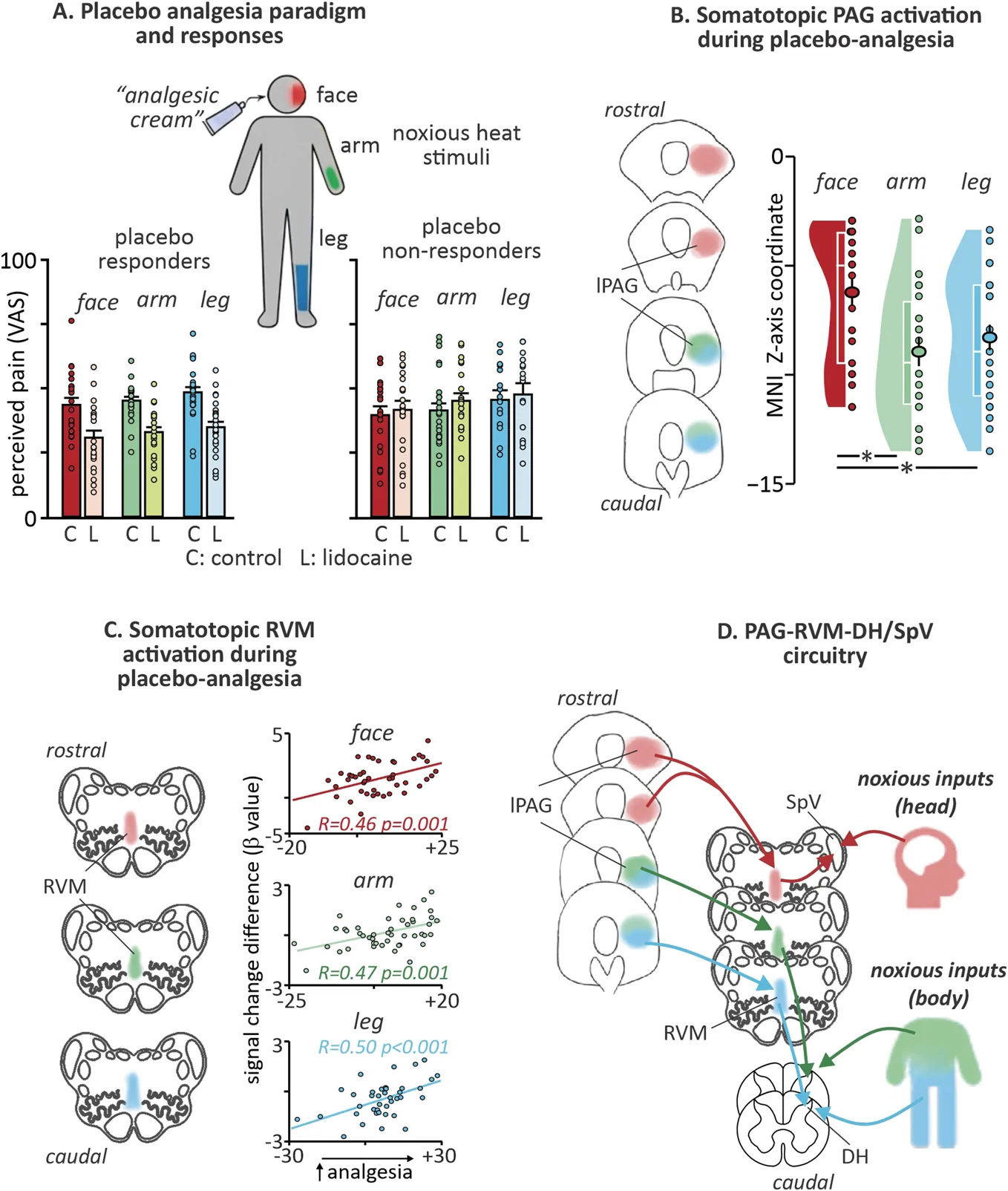
(A) Individual and mean ± SEM pain intensities evoked by noxious thermal applied to control and placebo (lidocaine) cream sites on the face, arm and leg. Participants were categorized as either responders or nonresponders. (B) Schematic representative coronal sections through the midbrain periaqueductal gray matter (PAG) showing the locations of maximal functional magnetic resonance imaging (fMRI) signal intensity changes in the during placebo hypoalgesia on the face, arm and leg. Plots to the right are individual placebo analgesia participant responder peak fMRI signal intensity changes plotted as Montreal Neurological Institute space (MNI) Z co-ordinate. Note that face hypoalgesia is associated with signal changes in the rostral lateral PAG (lPAG), whereas arm and leg hypoalgesia are associated with changes more caudally in the lPAG. (C) Schematic representative coronal sections through the medulla showing the locations of placebo hypoalgesia-related signal intensity changes in the rostral ventromedial medulla (RVM). Note the rostrocaudal organization from face to arm to leg. Plots to the right are hypoalgesia against signal intensity changes; modified from 36. (D) Schematic representation of somatotopically organised brainstem analgesic circuitry. Noxious inputs from the head synapse in the spinal trigeminal nucleus (SpV), which in turn projects to the rostral lPAG. The rostral lPAG sends afferents to the rostral RVM, which can modulate SpV activity. By contrast, noxious inputs from the body synapse in the dorsal horn of the spinal cord (DH), which in turn projects to the caudal lPAG. The caudal lPAG sends afferents to the caudal RVM which can modulate DH activity.

Consistent with our hypothesis, analysis of fMRI activation patterns in placebo responders compared with nonresponders revealed a crude somatotopic organization within the PAG. Placebo hypoalgesia on the face evoked signal intensity change differences in the rostral lPAG, whereas placebo hypoalgesia on the arm or leg evoked signal differences more caudally in the lPAG (Fig. 3B). Consistent with this organization, linear regression analysis of differences in signal intensity change (control vs “lidocaine” scans) vs analgesic response magnitude also revealed the same somatotopic pattern. Importantly, we found that the noxious stimuli activated the contralateral lPAG, whereas placebo hypoalgesia preferentially recruited the ipsilateral lPAG. However, this apparent lateralisation should not be interpreted as indicating strictly unilateral analgesic output. The PAG is characterised by extensive bilateral interconnections between its columns, which support coordinated and often bilateral descending control of nociception.^41^ In this context, contralateral nociceptive inputs may initially engage primarily 1 side of the PAG, while placebo-related modulation may be driven by intra-PAG and top-down influences may recruit primarily the opposite side of the PAG. Ultimately, either side of the PAG likely contacts the RVM to modulate the RVM and DH/SpV bilaterally. This interpretation is consistent with the broader literature demonstrating predominantly bilateral analgesic effects following PAG stimulation.

Furthermore, in 23 of the participants undergoing testing for placebo responses in the face, we assessed the regional specificity of the placebo hypoalgesia response by testing pain intensity on the forearm site during face placebo. Comparison of pain intensity ratings between noxious stimulation of the skin site of the forearm revealed no significant reductions in pain intensity either within the whole group or in only those participants who showed a significant hypoalgesic response on the face. Our results indicate that hypoalgesic responses during the face placebo paradigm were restricted solely to the face and did not include the body. This spatial specificity is consistent with prior work demonstrating site-specific placebo analgesia. For example, a within-subject conditioning study applying placebo creams to different fingers showed that analgesic responses differed systematically between locations, such that the high-expectancy finger showed the greatest reduction in pain, the low-expectancy finger showed an intermediate reduction, and the control finger showed the least reduction, indicating clear site-specific and dose-dependent placebo effects.^118^

Our experiments also revealed a somatotopic organisation in the RVM, with face-related hypoalgesia associated with activity changes in the rostral RVM and body-related hypoalgesia associated with changes in the caudal RVM (Fig. 3C). These findings suggest that the rostral RVM neurons preferentially target the SpV, whereas caudal RVM neurons project preferentially in the DH. This is the first report of somatotopy within the RVM in humans and supports observations made in earlier preclinical studies for some degree of anatomical specificity in this region. For example, anterograde tracing studies show that midline RVM neurons (raphe magnus) project predominantly to deep DH laminae (V–VI), whereas more laterally positioned neurons (lateral paragigantocellularis) project to superficial laminae (I–II) of the DH.^60^ In addition, RVM projections to the DH are largely GABAergic and tend to contact neuronal somata, whereas projections to the SpV contain relatively few GABAergic neurons and preferentially target dendrites.^1^

Complementing this anatomical organisation, electrophysiological studies show that RVM neuronal properties depend on the source of afferent input. The classification of neurons into ON, OFF, and NEUTRAL cells varies with stimulation of spinal vs craniofacial, visceral, or vagal territories, suggesting that distinct afferent channels recruit partially segregated circuits within the RVM.^24^ In particular, trigeminal input alters the relative proportions of these cell types compared with spinal stimulation, reducing NEUTRAL cells and increasing ON and OFF populations, consistent with input-specific organisation.^90^ Together, these findings indicate that the RVM is not a uniform output structure but exhibits spatial and functional organisation that reflects the origin and modality of nociceptive input.

Our human data convincingly demonstrate that, as for the lPAG, the RVM exhibits a regionally specific, rostrocaudal somatotopic activation during placebo-mediated hypoalgesia. This lPAG-RVM organization supports our hypotheses of an integrated framework in which somatotopic organisation is embedded within broader functional specialisations, such that rostral and caudal lPAG subregions are differentially engaged in distinct defensive behavioural modes, with somatotopically targeted analgesia emerging as part of these behaviourally relevant circuit outputs (Fig. 3D). Whether the vlPAG is also somatotopically organised for deep/inescapable pain remains unknown. However, given that inescapable or deep pain drives almost identical passive defensive behaviours, characterised by quiescence and withdrawal, regardless of body origin, this degree of somatotopic organization seems somewhat redundant.

## 6. Periaqueductal gray and conditioned pain modulation analgesia

Consistent with the somatotopic organization of the lPAG, we have recently shown that another hypoalgesia paradigm, conditioned pain modulation (CPM), also involves lPAG recruitment. Conditioned pain modulation refers to the phenomenon where the perception of pain from 1 stimulus is reduced by the presence of a second, spatially separate noxious stimulus.^164^ Using ultra-high-field fMRI, we explored brainstem activation patterns during a CPM paradigm in 45 pain-free individuals.^153^ The CPM protocol involved an initial series of 8 noxious thermal stimuli applied to the face, followed by a second series of 8 noxious thermal stimuli on the face applied concurrently with tonic leg muscle pain elicited by an injection of hypertonic saline into the tibialis anterior muscle (Fig. 4A). We found that 18 participants showed a significant hypoalgesic response (responders) and 16 showed no significant change in pain intensity (nonresponders) during the CPM paradigm (11 participants displayed a hyperalgesic response). Exploring the relationship between pain intensity changes in responders and nonresponders with brainstem signal intensity changes, we found that CPM-hypoalgesia was associated with signal intensity changes in brainstem regions previously shown to be critical for CPM-like responses. These included the region of the subnucleus reticularis dorsalis and A5 cell group,^23,93^ and we also found signal intensity changes in the lPAG (Fig. 4B). These hypoalgesia-related signal changes were in the same direction as those evoked by placebo, ie, hypoalgesia associated with signal intensity decreases. Although we did not assess CPM responses in multiple locations and therefore cannot definitively conclude that CPM also recruits the lPAG in a somatotopic fashion, the rostrocaudal location of face CPM lPAG activation was within 1 mm of that observed for placebo hypoalgesia. Future studies are needed to confirm that a somatotopic organization of the lPAG also occurs beyond placebo hypoalgesia and occurs during at least 2 hypoalgesic paradigms.

**Figure 4.**
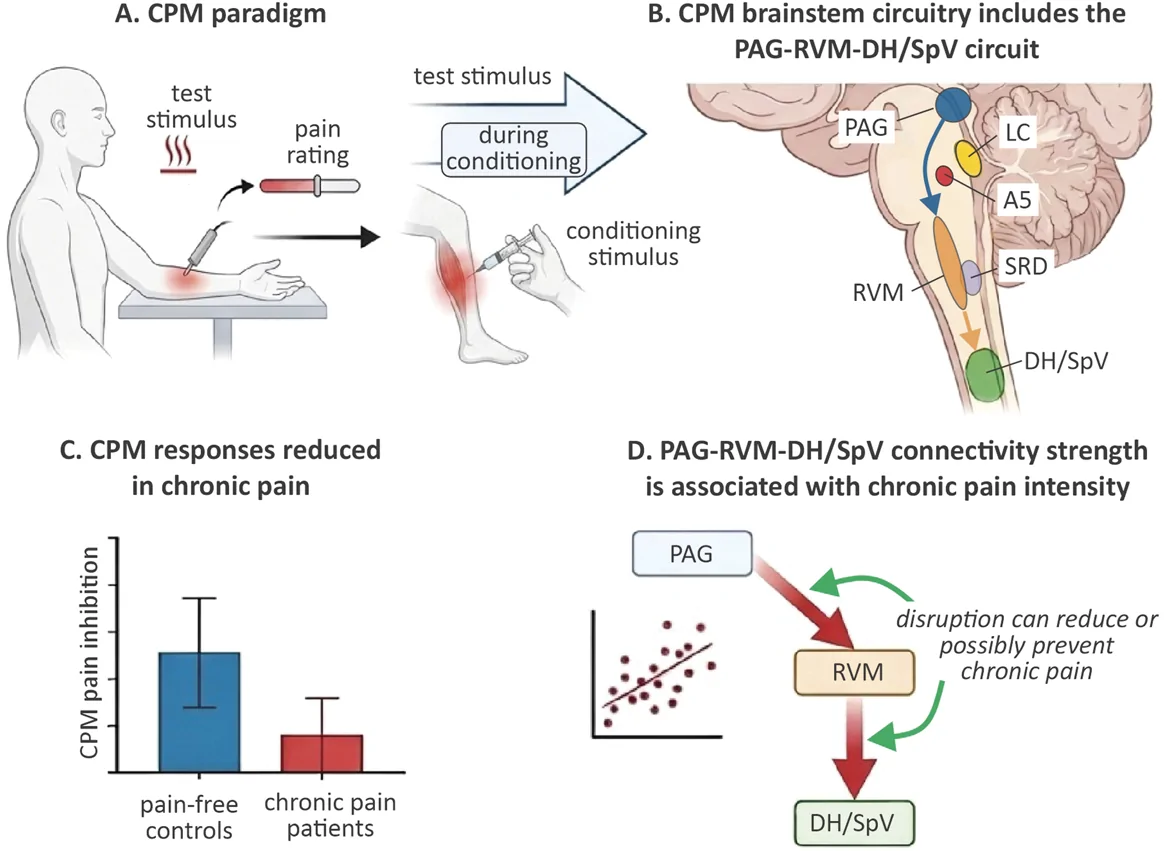
(A) Conditioned pain modulation (CPM) protocol involved a series of 8 noxious heat stimuli applied to the face (control). This was then repeated in the presence of a tonic noxious muscle stimulus applied to the leg (CPM). A significant decrease in mean pain intensity between the 8 noxious heat stimuli during the CPM compared with 8 noxious heat stimuli during the control period is the CPM hypoalgesic magnitude. (B) Location of brainstem regions involved in CPM responses. These include the midbrain periaqueductal gray matter (PAG), locus coeruleus (LC), A5 cell group, rostral ventromedial medulla (RVM) and the subnucleus reticularis dorsalis (SRD). These sites act directly or indirectly on the dorsal horn (DH) and spinal trigeminal nucleus (SpV) to modulate incoming noxious information. (C) There is evidence that the magnitude of CPM-hypoalgesia responses is significantly reduced in individuals with chronic pain, indicative of impaired endogenous analgesic circuitry function. (D) There is also evidence that signal covariation strengths in the PAG-RVM-DH/SpV circuit are altered in chronic pain and that disrupting this circuit can reduce the intensity or possibly prevent the development of chronic pain.

A key issue in interpreting these fMRI findings concerns the cellular and circuit-level substrates of the blood oxygen level-dependent (BOLD) signal within the PAG-RVM pathway. Importantly, evidence suggests that BOLD signals do not directly index the spiking output of projection neurons but instead reflect local synaptic and metabolic activity, including both excitatory and inhibitory processes.^74,98^ Within the PAG, a decrease in BOLD signal during analgesia may therefore reflect engagement of local inhibitory microcircuits rather than reduced functional output. One plausible mechanism is suppression of GABAergic interneurons within the PAG can reduce local synaptic activity while simultaneously increasing the activity of downstream projecting neurons to the RVM.^7,15,148^ In this framework, reduced PAG BOLD is compatible with enhanced descending drive. By contrast, the positive relationship between RVM BOLD signal and analgesia magnitude may more directly reflect increased antinociceptive output, potentially through enhanced activity of OFF cells and/or a shift in the balance away from pronociceptive ON cells.^39^ These apparently opposing BOLD directions are therefore not necessarily contradictory but may instead reflect different aspects of processing within the pathway, ie, local microcircuit modulation in the PAG vs net output at the level of the RVM. This interpretation is further consistent with prior work showing context-dependent PAG signals, such as increased activity during anticipation and stronger functional coupling with prefrontal regions, which likely reflect top-down excitatory input distinct from the local disinhibitory mechanisms engaged during sustained analgesia.^20,145,151,152^

The PAG is increasingly recognised not only as a descending modulatory structure but also as an integrative hub contributing to ascending signalling involved in prediction, learning, and cognitive control of pain.^58,97,156^ Human fMRI studies show that it encodes aversive prediction errors, receives value-related prefrontal inputs, and reflects negative prediction errors during omission of expected aversive outcomes, placing it within reinforcement- and safety-learning circuits involving PAG-medial prefrontal interactions.^62,129^ These findings align with predictive coding models in which pain emerges from integrating sensory input with expectations and prediction errors across distributed circuits. Thus, the BOLD signal likely reflects both descending modulation and expectancy-related computations, representing integrated circuit-level processing; the observed rostrocaudal and somatotopic organisation may therefore reflect coordinated engagement of specialised PAG subcircuits supporting nociceptive control and adaptive behaviour.

## 7. Periaqueductal gray and chronic pain

There is growing evidence that individuals with chronic pain exhibit impaired endogenous analgesic function. Specifically, a reduced capacity to regulate or dampen incoming nociceptive input appears to increase vulnerability to the development of chronic pain following injury.^72^ Evidence from both preclinical models and human research consistently demonstrates that chronic pain is associated with significant anatomical, biochemical, and functional changes at the level of the primary afferent synapse. These alterations include excitotoxic neuronal damage driven by sustained increases in afferent input.^6,116,133,160,161^ In addition, experimental animal studies indicate that chronic neuropathic pain is linked to neuroplastic changes within brainstem pain-modulatory systems.^71,125^ Such adaptations alter DH/SpV output, contributing to both the initiation and persistence of hypersensitivity,^102,123,155^ as well as broader behavioural disturbances.^22,117^ Consequently, individuals who can effectively recruit descending inhibitory pathways may be better able to limit DH/SpV sensitisation and thereby reduce their risk of developing chronic pain.

Consistent with this, chronic pain conditions are frequently associated with altered responses to acute nociceptive stimuli and reduced efficacy of CPM.^38,96,163^ Impaired CPM has been documented across a wide range of chronic pain conditions, including migraine, arthritis, fibromyalgia, temporomandibular disorder, irritable bowel syndrome, and neuralgias^96,163^ (Fig. 4C). Furthermore, reduced CPM performance has been proposed as a risk factor for the development of persistent pain following surgery.^44,95^ There is also increasing evidence that CPM may help predict treatment outcomes. For example, reduced CPM efficiency has been associated with more favourable responses to duloxetine in individuals with painful diabetic neuropathy.^165^ Similarly, improvements in CPM have been observed alongside pain relief following interventions such as total joint replacement for osteoarthritis; pharmacological treatments including morphine, ketamine, and placebo for neuropathic pain; and pregabalin therapy in fibromyalgia.^92,119,162^

Together, these findings highlight the central importance of endogenous analgesic systems, particularly the PAG-RVM-DH/SpV pathway, in determining both susceptibility to chronic pain and responses to treatment. Despite this, the precise contribution of brainstem circuits to chronic pain remains incompletely understood, and no human studies have specifically isolated CPM-related changes in brainstem function. Nevertheless, preclinical and neuroimaging research has begun to characterise alterations within this circuitry. In animal models of neuropathic pain, vlPAG neurons exhibit altered excitability, as well as abnormal spontaneous and stimulus-evoked firing patterns that are associated with pain-related behaviours.^43,75,131^ Similarly, changes in RVM function are well documented, including altered activity of ON and OFF cells, reduced activation thresholds, and abnormal responses to both mechanical and thermal stimuli.^27,61,137^ Specifically, chronic pain models show decreased spontaneous activity of OFF cells alongside increased spontaneous firing of ON cells.^61,137^ Importantly, disruption of the PAG, RVM, or the RVM-DH pathway can reduce pain-related behaviours,^123,155^ and interference with RVM function prior to injury may even prevent their development^124^ (Fig. 4D).

Human neuroimaging studies provide converging evidence for altered function within these brainstem networks. Structural MRI investigations have identified volumetric and diffusion changes in the PAG, RVM, and SpV in individuals with chronic orofacial pain conditions, including trigeminal neuropathy, temporomandibular disorder, and migraine.^100,160,161^ Functional MRI studies further demonstrate altered connectivity within this pathway. For example, individuals with chronic orofacial neuropathic pain show increased functional coupling between the RVM and both the PAG and SpV compared with healthy controls^110^ (Fig. 4D). By contrast, patients with painful temporomandibular disorder display increased RVM-SpV coupling without corresponding changes in PAG-RVM connectivity, suggesting that different types of chronic pain may involve distinct patterns of brainstem dysfunction.^108^ Moreover, dynamic fluctuations in connectivity within the PAG-RVM-SpV pathway have been shown to track spontaneous changes in pain intensity, with stronger coupling observed during periods of higher reported pain.^109^

In addition to the PAG-RVM-DH/SpV pathway, other brainstem regions, including the locus coeruleus, have also been implicated in chronic pain mechanisms. Preclinical studies report mixed findings, with some suggesting that the locus coeruleus exerts inhibitory influences on DH activity following nerve injury,^68,80^ while others indicate a facilitatory role in maintaining altered sensory processing and affective disturbances.^22,85^ Notably, ablation of noradrenergic neurons has been shown to reduce mechanical hypersensitivity for extended periods.^85^ In humans, locus coeruleus activity is associated with offset analgesia-mediated pain reductions, and increased functional connectivity between the RVM and both the locus coeruleus and the subnucleus reticularis dorsalis (SRD) has been observed in chronic neuropathic pain.^33,110^ However, the absence of altered connectivity between the locus coeruleus and SpV suggests that direct descending noradrenergic projections to the DH/SpV may play a limited role in maintaining chronic pain in humans.

The SRD has also been shown to contribute to pain modulation. In preclinical models, inhibition of SRD activity significantly reduces both spontaneous and noxious-evoked firing of DH neurons.^139^ Furthermore, noxious stimulation increases noradrenaline release within the SRD, and pharmacological blockade of this release attenuates both allodynia and hyperalgesia.^101,103^ These effects are unlikely to be explained by changes in baseline activity, as tonic firing in the locus coeruleus appears largely unchanged following nerve injury.^22,149^ In addition, reductions or complete loss of diffuse noxious inhibitory controls have been consistently observed after nerve injury.^10,122^

Overall, these findings collectively demonstrate that chronic pain is associated with significant reorganisation of brainstem pain-modulatory circuits. Such alterations likely enhance descending facilitation at the level of the primary afferent synapse, thereby increasing nociceptive transmission to higher brain centres. A more detailed understanding of how endogenous analgesic circuits are altered in chronic pain may provide important opportunities for therapeutic intervention, as targeting these pathways could help restore balanced pain modulation and reduce persistent nociceptive signalling.

## 8. Conclusion

Endogenous pain modulation is an evolutionarily conserved function embedded in brainstem circuitry, with the PAG-RVM-SDH/SpV pathway serving as a core substrate for context-dependent suppression or facilitation of nociception. Distinct PAG columns support different defensive strategies, forms of hypoalgesia, and autonomic-behavioural outputs, highlighting tight links between pain control and survival. Ultra-high-field fMRI extends this to humans, showing conserved PAG organisation, including a coarse rostrocaudal somatotopy in lateral PAG that aligns noxious inputs with spatially matched hypoalgesia. Placebo and conditioned modulation recruit this organisation, while parallel somatotopy in the RVM suggests anatomically matched modules enable region-specific descending control. Together, these findings indicate that hypoalgesia is dynamic, behaviourally appropriate, and spatially targeted rather than global. Disruption of this system may contribute to chronic pain, and restoring its organisation offers a promising avenue for targeted, mechanism-based therapies.

## Conflict of interest statement

The authors have no conflict of interest to declare.
